# Patients’ experience and satisfaction with GP led walk-in centres in the UK; a cross sectional study

**DOI:** 10.1186/1472-6963-13-142

**Published:** 2013-04-18

**Authors:** Mubashir Arain, Jon Nicholl, Mike Campbell

**Affiliations:** 1 (ScHARR) School of Health and Related Research, The University of Sheffield, Sheffield, UK; 2 Professor of Health Services Research, The University of Sheffield, Sheffield, UK; 3 Professor of Medical Statistics, The University of Sheffield, Sheffield, UK

**Keywords:** Health services, Urgent care services, Primary care service, Health care centre, Walk-in centres, NHS, England

## Abstract

**Background:**

GP led walk-in centres were established in the UK in 2009. Around 150 such clinics were initially planned to open. Their purpose is to provide a primary health care service to complement the urgent care services provided by Emergency Departments (ED), to reduce unnecessary patient attendance at ED, and to increase accessibility of health care services. The objectives of this study were to determine patient satisfaction and experiences with GP led walk-in centres in the UK.

**Methods:**

A survey was conducted in two GP led walk-in centres in the North of England over three weeks during September and October 2011. A self reported, validated questionnaire was used to survey patients presenting at these centres. A short post visit questionnaire was also sent to those who agreed. Ethical approval for the study was obtained from an NHS ethical review committee.

**Results:**

Based on a sample of 1030 survey participants (Centre A = 501; Centre B = 529), we found that 93% of patients were either highly or fairly satisfied with the service at centre A and 86% at centre B. The difference between centres was due to the longer reported waiting times which were seen in centre B. There was no difference in satisfaction between first time users and repeat users (P value = 0.8). Roughly 50% (n = 507) of patients reported that their reason for using the walk-in centre was having GP access without an appointment, and 9% (n = 87) reported that their GP surgery was closed. A further 20% of patients (n = 205) reported that they were not able to see their own GP because of their working hours.

In the post visit survey (n = 258), nearly all patients reported complying with the advice given (around 90% at both study centres), and most of the patients (86%) reported their problem had resolved a few days later. In addition, 56% of patients at centre B and 58% at centre A reported that they had also visited another NHS service for the same problem, mostly their own GP (66%).

**Conclusions:**

The GP led walk-in centres increased access to GP care and most of the patients were satisfied with the service.

## Background

General Practitioner (GP) led walk-in centres were established in England in 2009 to increase GP accessibility and to decrease unnecessary attendance at Emergency Departments (EDs). The centres were set up after a report by the Department of Health which identified a need to improve accessibility to urgent care services [1]. Each primary care trust (PCT) was expected to set up one centre to cover the needs for the residents to see a GP without prior appointment in case of urgent need. The GP led walk-in centres aimed to improve health care accessibility for the general public by making GPs available during evening times as well as at weekends. The centres provide a number of health care services such as nurse practitioner consultation (and GP consultation if needed), repeat prescriptions, vaccination services, and health care advice. The centres are able to refer patients to the Emergency Department in case of a serious problem.

Nurse led walk-in centres were first established in the UK in 2001, and consultations were predominantly provided by nurses (nurse practitioners, advanced nurse practitioners, or consultant nurses). One postal survey of GPs revealed that around one third believed that walk-in centres increased patients’ expectations, and they were also concerned with continuity of treatment and patient safety [2]. There was also a concern about the need for better communication between these service providers and the registered GPs. On the other hand, another survey showed that patient satisfaction with the quality of service was greater in walk-in centres as a result of easy access and much shorter waiting times as compared to GP practices [3]. Recently, Australia also adopted the model of nurse led walk-in centres and some of the centres are already functional [4]. However, a recent paper suggested that these walk-in centres would be more beneficial if there was a doctor available [4]. In Canada, walk-in clinics are mostly run by doctors and studies have shown a high satisfaction with these clinics [5].

In the UK, Primary Care Trusts are responsible for the quality and accessibility of primary health care services. Most primary care is delivered by GPs who are responsible for 24 hours care. However, GPs can opt out of providing 24 hours care, and can delegate this to GP out-of-hours services which typically operate from 6pm to 8am during week days and for 24 hours at weekends [6], and are staffed by nurses and GPs. Alongside the GP service, most areas now have walk-in centres led either by nurses or by GPs where patients can turn up without an appointment. Other than a GP, patients can see a pharmacist or call NHS Direct (a national telephone based service), in addition to the option of visiting an ED. EDs in the UK (formerly known as Accident and Emergency Departments) can deal with all urgent health problems and most are open 24 hours/day. However, EDs are not recommended to be used if a problem is not urgent or can be managed in a primary health care setting.

GP led walk-in centres in the UK provide nurse and GP consultations, and hence are expected to address some of the above concerns about nurse led walk-in services, where consultations are only provided by nurse practitioners. We have sought to determine patients’ experiences and satisfaction with GP led walk-in centres to help clarify the role of these services in improving primary health care provision.

Patients’ experiences with a service are a self reported record of different aspects of the processes of care experienced while using a service such as how accessible the service was for the patient, the waiting time, and the availability of appointments [7]. On the other hand, a patient’s satisfaction with a service represents their response to those experiences and this may be directly related to their prior expectations and a number of other factors which can influence the satisfaction level. Patients’ reported experiences are considered to be less subjective than their reported satisfaction [8] and a patient may be satisfied with a service, although the reported experience was suboptimal [9]. So although there is usually a significant association between patient experiences and global satisfaction with a service [10], it is recommended that patient experiences with the service rather than satisfaction should be used for monitoring purposes [11]. We expected that a survey questionnaire including both patient experience questions as well as questions related to satisfaction would provide a better understanding about the quality of the service than questions about either alone. However, the main focus of this paper is patient satisfaction with the GP led walk-in centre services.

We aimed to survey patients attending GP led walk-in centres to identify whether they addressed the patients’ needs. In addition, we were interested in looking at the impact of the opening of GP walk-in centres on ED services. We conducted two surveys, one on site, and the other a post-visit survey 4 weeks after visiting the GP walk-in centres to determine whether or not patients had had to use another NHS service for the same problem. This paper is about patient satisfaction and experience with the service.

## Methods

We conducted a mixed method evaluation that consisted of a cross-sectional survey, ED routine data analysis, and qualitative interviews. However, this paper only presents the first component of the study which is the cross-sectional survey of patients, a subsequent paper will report the impact of the GP led walk-in centre on the NHS.

Patients were enrolled from two GP walk-in centres in the North of England (Centre A and Centre B). The two centres represent two different models of GP walk-in centres. Centre A was built in a community health centre alongside a number of other primary care services such as a sexual health service, physiotherapy, a diagnostic laboratory and a pharmacy. Centre A was in a large town with a population of around 250,000. Centre B was built as an independent GP led walk-in centre which provides consultations for minor health problems, and is located in the centre of a large student city with a population of around 500,000.

The survey was conducted from September 2011 to October 2011 at these GP walk-in centres. A self-report, validated questionnaire [Additional file 1: Appendix I] was used to determine what kind of patients used these services, how satisfied they were with the location and opening hours of the centre, what they would have done in the absence of the GP walk-in centre service, their experience of waiting, satisfaction with the service, and referral information to other NHS services. For a child patient, the questionnaire was filled-in by the accompanying adult. The questionnaire was originally developed and piloted in 2002 by Salisbury et al. [3] for evaluating nurse led walk-in centre services and their validity data showed a high level of internal consistency for different questions. Another study used the same tool with some amendments to enable it to be used for commuter walk-in centres [12]. The satisfaction scale used in this study was exactly the same as used in these previous studies [3,12] to enable comparisons to be made between different models of care. The questionnaire was not re-validated in this study. There were some differences in other questions such as location of the centre but the satisfaction scale used in all these studies was the same. All patients who presented to the centres during the study period were potential study participants and the only patients excluded were those in whom language was a barrier since the questionnaire was only available in English. Patients with serious health conditions were offered the survey questionnaire but clearly informed that the questionnaire could be filled-in later on and posted using a prepaid envelope provided.

We aimed to sample at least 400 patients from each centre to obtain statistically robust estimates of the proportions of patients reporting characteristics such as satisfaction with care. This sample size was calculated in order to estimate the proportions of patients reporting dichotomous outcomes with 95% confidence intervals of less than +/− 5%.

The survey questionnaire was distributed by receptionists at the centres. The receptionists aimed to distribute the survey questionnaire to every consecutive patient attending the walk-in centre during the survey period. Questionnaires were also placed near the reception for patients to take if the receptionist was not able to hand them over during very busy times. We placed a box near the reception for patients to drop-in completed questionnaires. We also provided self addressed, prepaid envelopes for patients to return the questionnaire by post if they preferred. Three nominal prizes were offered at each centre for patients randomly selected from those who returned a questionnaire and agreed to participate in the draw.

We also offered to send respondents a short post visit questionnaire [Additional file 2: Appendix II] to ask if they had used another NHS service after visiting the walk-in centre for the same problem. The post visit questionnaire also enquired about compliance with the treatment or advice given at the centre and whether or not the problem was fully or partially resolved or not resolved at all. The post visit questionnaire was sent 3–4 weeks after the visit, along with a self-addressed prepaid envelope.

Data was entered and analyzed in PASW statistics 18. Logistic regression on a dichotomised patient satisfaction variable was used to estimate the influence of different factors on the satisfaction of patients with the service. The model was developed using all the factors available which were likely to influence patients’ satisfaction. Those variables which appeared to be significantly associated with the satisfaction such as age, location, and waiting time, were inserted into the final model to explain any difference in satisfaction between centres, after controlling for any confounding effects. Chi-square and t-test were applied for categorical and continuous data respectively. Frequencies, means and ranges are also reported in the tables where appropriate.

Ethical approval of the study was obtained from an NHS ethical review committee (REC reference number 10/H1304/31). Every patient received a patient information sheet, approved by the NHS ethics committee and the project was reviewed by the Consumers Research Advisory Group (CRAG). We reported back the results to each centre to explain our findings and obtain their feedback. We also retrieved routine data from each centre to compare with our respondents to establish whether the survey participants were a true representative sample of the patients attending these GP walk-in centres. We compared the mean age and the age distribution, sex, and time of presentation at the centre between those who responded to our survey and the routine data to help validate our results.

## Results

**Figure 1:**
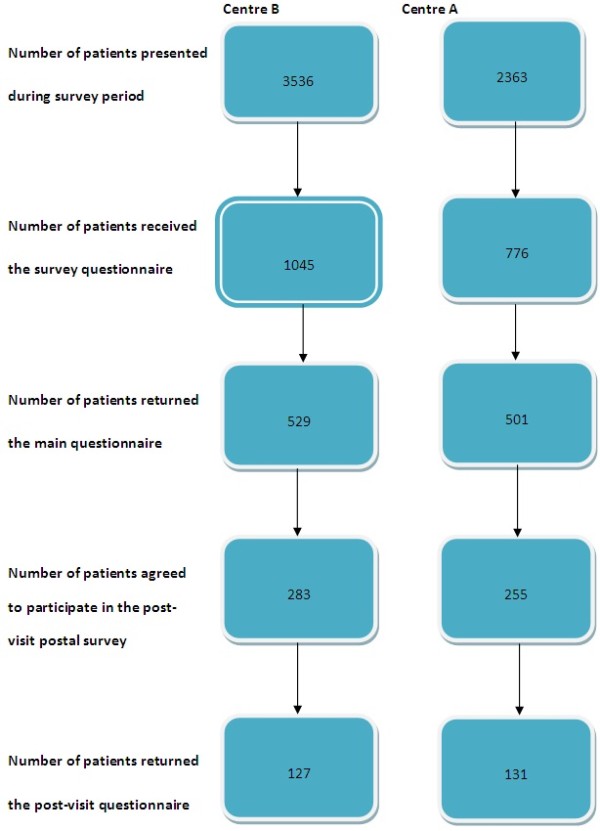
Response of participants to the main survey and post-visit survey.

A total of 1030 patients participated in the study (response rate 57%), 529 from centre B (response rate 51%) and 501 from centre A (64%) [Figure 1]. A majority of patients were female (59%) and the mean age of the patients was 31.5 years (range = 0 – 89yrs) [Table 1]. The survey sample was compared with the centres’ routine data to examine the representativeness of the sample and no significant difference was found for age distribution, sex, or time of attending the centre. However, the respondents at Centre A were in higher proportion of those who attended the service during evenings and weekends.

**Table 1:** Characteristics of patients presenting at the GP walk-in centres

**Demographic characteristics**	**Centre B**	**Centre A**	**Total**
	**n = 529**	**n = 501**	**n = 1030**
Age (years) Mean ± SD, (Median) [Range]	32.1 ± 17.9,	30.9 ±21.7,	31.5 ± 19.8,
	(27) [0, 82]	(28) [0, 89]	(27) [0,89]
Sex % (n)			
Male	37.5 (188)	39.5 (209)	38.5 (397)
Female	58.7 (294)	58.8 (311)	58.7 (605)
Missing	3.8 (19)	1.5 (9)	2.5 (28)
First time user of the GP centre % (n)	52% (272)	33% (164)	43% (436)
Occupation	% (n)	% (n)	% (n)
Working full-time	34.8 (184)	35.3 (177)	35.0 (361)
Student	28.2 (149)	17.2 (86)	22.8 (235)
Working part-time	10.2 (54)	8.0 (40)	9.1 (94)
Retired	8.5 (45)	8.4 (42)	8.4 (87)
Unemployed	7.8 (41)	9.2 (46)	8.4 (87)
Pre-school infants	2.3 (12)	11.8 (59)	6.9 (71)
Homemakers	0.8 (4)	1.8 (9)	1.3 (13)
Other	6.0 (32)	3.8 (19)	5.0 (51)
Missing	1.5 (8)	4.6 (23)	3.0 (31)

Most of the patients used the centres as a walk-in service and only 4% of patients had made a prior appointment. 50% (n = 507) of patients reported the reason for coming as having quick GP access without an appointment and an additional 9% (n = 87) reported their GP surgery was closed. A further 20% of patients (n = 205) reported that they were not able to see their own GP because of their working hours, and 5% (n = 54) were not registered with any GP. There were a significantly higher proportion of unregistered patients at centre B (n = 44) as compared to centre A (n = 10); P value < 0.001. About 13% (n = 131) reported shorter waiting times than in ED as a reason for coming. Only 4% (n = 33) were referred from other NHS services. Around 65% (n = 548) of patients presented during out-of-hours periods.

At centre A most of the patients (64%) were highly satisfied overall with their visit to the service and a further 30% reported themselves to be fairly satisfied [Table 2]. The mean satisfaction score was 4.5 on a five point Likert scale. Most patients rated the opening hours as excellent (41%) or very good (52%), without any significant difference between first time attendees and those who had use the service before (P value = 0.6); 35% rated the centre’s location as excellent and 53% rated it as very good [Table 3]. 54% of the patients were seen by a GP only, 38% by a nurse practitioner only, and 8% by both a GP and a nurse. The patients’ reported mean waiting time was 40.9 minutes (SD = 32mins, range 3mins to 240mins) with a small difference between office hours and out-of-hours (evenings and weekends), [office hours = 33.5, SD = 27.4 versus out-of-hours = 42.2mins, SD = 32.3; P value = 0.06].

**Table 2:** Patient satisfaction scale (Rating 1–5), comparison between the centre A and B

**Survey questions**	**Centre B**	**Centre A**	P-value*
	**Mean (SD)**	**Mean (SD)**	
Attitude of receptionist	4.4 (0.87)	4.6 (0.67)	<0.001
	n = 466	n =449	
Time you had to wait before seeing a nurse or doctor	3.2 (1.34)	4.0 (1.13)	<0.001
	n =449	n =431	
Attitude of nurse or doctor	4.6 (0.79)	4.7 (0.67)	0.07
	n =439	n =434	
Satisfaction with the explanation about problem by doctor or nurse	4.4 (0.88)	4.6 (0.7)	0.02
	n =430	n =434	
Treatment or advice	4.4 (0.9)	4.5 (0.78)	0.06
	n =430	n =435	
Overall satisfaction with the service (for this visit)	4.3 (0.97)	4.5 (0.78)	<0.001
	n =439	n =436	
Overall satisfaction distribution (%)	4.3 (0.97)	4.5 (0.78)	<0.001
	n =430	n =435	
Rated 5 Very satisfied	49.0%	63.5%	-
Rated 4 Fairly satisfied	37.1%	29.8%	-
Rated 3 Uncertain	7.1%	3.0%	-
Rated 2 Not very satisfied	3.4%	2.3%	-
Rated 1 Not satisfied at all	3.4%	1.4%	-

* P values are obtained after controlling the effect of other relevant factors including age, sex, ethnicity, office hours or out-of-hours, first time user.

**Table 3:** Comparison of responses between the patients of the Centre A and B on the main survey at the centre (n = 1030)

**Survey questions**	**Centre B**	**Centre A**	P-value*
	**n = 529**	**n = 501**	
Convenience of the location mean Likert scale (1–5) score (SD)	3.9 (0.80)	4.2 (0.67)	<0.001
Convenience of the opening hours mean (SD)	4.3 (0.67)	4.3 (0.64)	0.58
Patients’ reported waiting time in minutes mean (SD)	74.2 (49)	40.9 (32)	<0.001
Expressed intention to visit another service for the same problem % (n)	24.7% (108)	24.3% (106)	0.47
First time user of the GP walk-in centre % (n)	52% (272)	33% (164)	<0.001
Would you use this walk-in centre again			<0.001
Rated 5 Definitely Yes	51% (222)	67% (292)	
Rated 4 Probably Yes	37% (164)	27% (119)	
Rated 3 Uncertain	7% (30)	4% (16)	
Rated 2 Probably Not	4% (16)	1% (5)	
Rated 1 Definitely Not	2% (8)	1% (4)	

* P values are obtained after controlling the effect of other relevant factors including sex, age, ethnicity, office hours or out-of-hours attendance.

At centre B, 49% were highly satisfied with the overall service provided at the centre and the mean satisfaction score was 4.2 on the Likert scale, which was significantly lower than the average satisfaction score at centre A. However, the difference was not significant after controlling for the effect of waiting time. Most of the patients were either highly satisfied (39%) or fairly satisfied (51%) with the opening hours of centre B. 25% reported the location of the centre as excellent and 52% reported it as a good, but those who had used the service before were more likely to report the convenience of the centre’s location as excellent (score = 5) than first time attendees [32% vs 19%, P value = 0.01]. 38% of the patients at centre B centre were seen by a nurse practitioner only, 35% by a GP only, and 26% by both a GP and a nurse. The reported mean waiting time for the patients was 74mins (SD = 49mins) with a significantly longer duration during evenings and weekends as compared to office hours on week days [85.1mins, SD = 54.3 vs 62.4mins, SD = 39.4; P value <0.001]. The waiting time was also significantly higher for those seen by two health care professionals as compared to those seen by a GP or a nurse practitioner only [85.3mins, SD = 53.3 versus 69.5mins, SD = 47.4; P value <0.001].

The post-visit, postal questionnaire asked about compliance with the treatment or advice given, resolution of the problem and whether or not the patient had had to visit another NHS service [Table 4]. Most of the patients (90%) followed the treatment/advice completely and there was no significant difference between the centres regarding the patients’ reported compliance for the treatment/advice. Similarly, there was no difference between the responses of patients about resolution of their health problem after visiting the centre. In addition, 56% of patients at centre B and 58% at centre A reported that they had also visited another NHS service for the same problem, mostly their own GP (66%). Those who did not use any other service were more likely to report that their health problem was fully resolved than those who had had to use another service for the same health problem (84% versus 25%, P value <0.001).

**Table 4:** Comparison of responses between the patients attending centre A and B who responded on the post-visit, postal survey (n = 258)

**Survey questions**	**Centre B**	**Centre A**	**Chi**^**2 **^**statistics**	**P-value**
	**% (n)**	**% (n)**		
Follow the advice (treatment)				
Completely followed	90 (113)	90 (118)		
	9 (12)	8 (11)	0.39	0.8
Partially followed				
	1 (1)	2 (2)		
Not followed				
Health problem solved				
Completely resolved	61 (78)	57 (75)		
Partially resolved	24 (30)	32 (42)	2.80	0.3
Not resolved at all				
	15 (19)	11 (14)		
Visited another NHS service after visiting the walk-in centre	39.7 (56)	41.4 (58)	0.09	0.81

Table 5 shows the regression model for the patient’s satisfaction variable. The satisfaction scale was dichotomised into highly satisfied (scored 5 on the Likert scale) and not highly satisfied (scored 4 or less on the Likert scale). The most significant factor affecting satisfaction, responsible for the difference in the patients’ satisfaction at the two centres, was the patient reported waiting time. After inserting this factor into the model there was no statistically significant difference in the satisfaction levels between the two centres. The other significant variable was the age group which showed that the least satisfied were those aged 15-24years and the most satisfied were those aged above 65years. The convenience of the GP walk-in centre’s location had also a significant association with the overall satisfaction.

**Table 5:** Logistic regression of explanatory variables against outcome of being “Highly Satisfied”

**First model, all variables**	Adjusted odds ratio* (95% CI)
Centre: A	1
B	1.1 (0.71 to 1.66)
Office hours	1
Out-of-hours	1.1 (0.76 to 1.72)
First time user	1
Used the centre before	0.84 (0.58 to 1.21)
Patient reported waiting time (mins)	0.98 (0.97 to 0.98)
Convenience of the location mean Likert scale (1–5) score	1.8 (1.26 to 2.43)
Seen by one health care professional	1
Seen by more than one health care professional	0.9 (0.58 to 1.61)
**Sex**	
Male	1
Female	1.1 (0.74 to 1.54)
**Age Group**	
0 – 15	1
16 – 24	0.4 (0.24 to 0.75)
25 – 44	0.8 (0.48 to 1.41)
45 – 64	1.4 (0.77 to 2.46)
65 +	3.4 (1.36 to 8.46)

*Adjusted for other variables in model.

## Discussion

This study provides the first evidence in the UK about patient satisfaction and experiences of GP led walk-in centres. The GP walk-in centres operate with longer opening hours than routine GP surgeries and open during weekends and bank holidays, have a GP at the centre along with nurse practitioners, and are able to retrieve patients’ records to update any treatment or advice given at the centre [13]. The location and opening hours of these centres are highly satisfactory for the majority of the patients. Convenience of centre A was reported as slightly higher, possibly because of the availability of free onsite car parking for patients. Studies have shown that patients use walk-in facilities because of easy access and much shorter waiting times as compared to GP practices [3]. Unregistered patients were in higher proportion at centre B, possibly because of the higher number of students living in the location. We found no major difference in satisfaction levels with this service between registered and unregistered patients.

Our study has sought to understand more about community needs and satisfaction with walk-in facilities. This is important because some of these centres have been closed because of lack of evidence of having any beneficial effects for the NHS [14]. Studies have shown that patients prefer to see a GP for unscheduled care instead of using other parallel services [15]. A large proportion of patients presented to the GP led walk-in centre because they were unable to reach their own GP (either the GP surgery was closed or the patient’s working hours did not allow them to see a GP) and in some cases they were not registered with any GP at all. In these circumstances, the patients would either present at ED, wait for their own GP, or may have just ignored their health problem, which could possibly have led to presenting at ED at a later time. Some GP led centres are now co-located with traditional nurse led walk-in centres. Studies have shown high patient satisfaction with nurse led walk-in centres in the past [3,16]. Thus, the model of combining two services, a nurse led walk-in centre and a GP centre, could be more effective than completely replacing one service with another.

We found that a high proportion of patients attending the two centres we have studied were very satisfied overall with the services. This was true for both first time users and repeat users and so is not just a type of ‘survivor’ effect due to dissatisfied patients subsequently using alternative services such as minor injuries units or ED. The satisfaction scale was dichotomised into “Highly satisfied” (score = 5) and “Not highly satisfied” (scoring 1–4) which is recommended as the most appropriate cut off for understanding patients’ satisfaction [17]. The longer time to be seen at one centre, particularly during evenings and weekends, was of concern. This also affected patients’ satisfaction with the service. The results reported in Table 5 show that the odds of reporting to be “highly satisfied” with the service reduce by around 2% with every minute increase in the waiting for treatment. After controlling for the effect of the waiting time there was no difference in the satisfaction level between the two centres. Studies show that waiting time is one of the important factors for evaluating emergency care services as it has significant impact on the quality of care and patients’ outcome [18,19]. Another study has reported that waiting time is a very important determinant of satisfaction in primary care out-of-hours services [20]. Patients seen by both a nurse and a GP had longer waiting times than those seen by one health care professional only. It was also observed in the analysis that the mean satisfaction score was significantly higher for those seen by one health care professional in comparison with two or more (Mean = 4.43, SD = 0.83 versus Mean = 4.22, SD = 1.02; P value = 0.02). In addition, the proportion of “Highly Satisfied” were also higher in those who were seen by one health care professional (58%) in comparison with two or more (49%) [Chi^2^ = 3.5; P value = 0.06]. However, after controlling for waiting time, there was no significant difference between the two groups. Centre B had a significantly higher proportion of patients seen by two health care professionals. The triage system at the two centres works differently, which might be responsible for the difference.

Previous studies have shown higher satisfaction rates with nurse led walk-in centres (79% reporting being highly satisfied) compared to the GP led walk-in centres we have studied (49% and 64%), though our results are comparable with reported patient satisfaction with GP practices (66%) [3]. The patient satisfaction levels we observed were also generally lower than those reported for nurse led commuter walk-in centres in London and outside London which ranged from 51% to 79% [21].

Our results show that most of the patients had very high compliance with the treatment/advice given at the centre and a large proportion of patients reported that their problem was fully resolved after visiting the centre. This suggests that the centres are important in fulfilling local community needs particularly at times when other services are not accessible. Our data also shows that the activity of these centres is higher at evening and weekends than during office hours, and this is one of the signs of increasing patients’ accessibility to GPs at times when their own GP is not available. However, it was also observed that a high proportion of patients visit their GPs soon after visiting the walk-in centre which suggests there is a risk of potentially duplicating the existing services. Though it would still be a useful service if patients would have otherwise gone to ED in times when their own GP was not opened. It was asked in the questionnaire that what patient would have done if the GP walk-in centre had not been established, which showed that around 23% of patients would have gone to ED; the proportion was higher (27%) for those who attended the service during evenings or weekends than those who attended during office hours (15%).

There are a number of important limitations to this study. First, we have only looked at two centres in the UK. We believe that the services offered by these two models are typical of others across the NHS, but it is possible that their locations and patient populations are not. Most of the other GP walk-in centre services in the UK would be similar to one model or the other or lie somewhere between these two models. The core purpose of the GP walk-in centres is identical all over the UK, which is to offer GP access without appointment and available over weekends and evenings. Therefore, the findings of this study can be used to understand satisfaction and experiences with GP walk-in centre in the UK. Walk-in Centres have been established in the United States, Canada [5] and also introduced recently in Australia [4]. In countries where services have just started or are being planned, it is very useful to refer to experiences with similar services in other countries. Thus, it is important to understand how these kinds of services work, what kind of patients attend these services and how effective they are in addressing patient needs. We believe the findings of this paper can be extrapolated to other similar settings where GP access needs to be improved.

Second, the response rate to our patient survey was only 57%, and the response rate to our follow-up post visit survey only 50% of those who received the questionnaire. Furthermore only around one third of the patients attending these services during the survey period received the questionnaire (an estimated 1821 out of 5899). In many surveys, the response rate is a major source of bias [22]. There were a number of reasons why the questionnaire was not received by every patient attending the service. Firstly, the survey questionnaire was handed out by the receptionists, so during some very busy hours it was not always possible to hand over the questionnaire to every single patient due to the time required to describe the study. Secondly, the questionnaire was given to the patients along with the patient registration sheet which every patient receives when they present to a walk-in centre. If the patient returned their completed registration sheet along with a non-completed questionnaire, the receptionists sometimes redistributed the questionnaire to the next patient. In this case it was not possible to keep a record of how many patients actually received the questionnaire. In addition, survey questionnaires were also placed in the waiting area to be accessible for every patient. Therefore, our estimates of the numbers receiving the questionnaire are based on the number of questionnaires known to have been distributed and the number of filled-in questionnaires returned to us. However, the number of patients who actually received a questionnaire may be larger than this. Studies have reported that patients’ satisfaction systematically differ between patients with different characteristics including age, sex and ethnicity [9,23]. However, the comparison of the demographics of our survey respondents with routine centre data did not show any significant difference between the two populations, so we expect that the sample is a true representation of the population.

Another limitation was the lack of recording the perceived health status of the patients in the survey. It has been reported that perceived health status is an important determinant of patient satisfaction [10,24]. Therefore, it could have been incorporated to help explain differences in satisfaction levels for example between centres or age groups. Lastly, the questionnaire was not re-validated for the purposes of this study, although the satisfaction scale used in the study was exactly the same as used in previous studies (3,12). It is possible that some of the dimensions of satisfaction with these services are missing in this scale. However, in this paper the analysis was based on “overall satisfaction” which includes all dimensions of satisfaction. There is a systematic review which has questioned the reliability and validity of questionnaires used to measure satisfaction with out-of-hours health care services [25]. The review found that most of the published satisfaction questionnaires are not fully validated to measure satisfaction and need to be used with caution. The review, however, suggested that it is preferable to use published scales rather than those which have not been published. Thus, the use of the same satisfaction scale in this study which has been used in similar health care settings by other studies enabled us to make comparisons with other satisfaction studies.

## Conclusions

In summary, GP led walk-in centres work to increase health care accessibility and a large majority of their patients are satisfied with the service. Most patients follow the advice they are given and their problems are resolved, though many subsequently use other NHS services so the impact on the local health economy remains questionable.

## Abbreviations

ED: Emergency department; PCT: Primary care trust; NHS: National Health Services; GP: General Practitioner

## Competing interests

The authors declare that they have no competing interests.

## Authors’ contribution

MA, JN and MC designed the project. MA collected data and prepared draft manuscript. JN and MC helped in data analysis. All authors reviewed the final draft.

## Pre-publication history

The pre-publication history for this paper can be accessed here:

http://www.biomedcentral.com/1472-6963/13/142/prepub

## Supplementary Material

Additional file 1 **Appendix I.** Main Survey Questionnaire. Click here for file

Additional file 2 **Appendix II.** Post visit questionnaire. Click here for file
