# Supplementary figures and images for: Patients’ experience and satisfaction with GP led walk-in centres in the UK; a cross sectional study
Source: BMC Health Serv Res. 2013 Apr 18;13:142. doi: 10.1186/1472-6963-13-142 (PMC3637583; doi:10.1186/1472-6963-13-142)

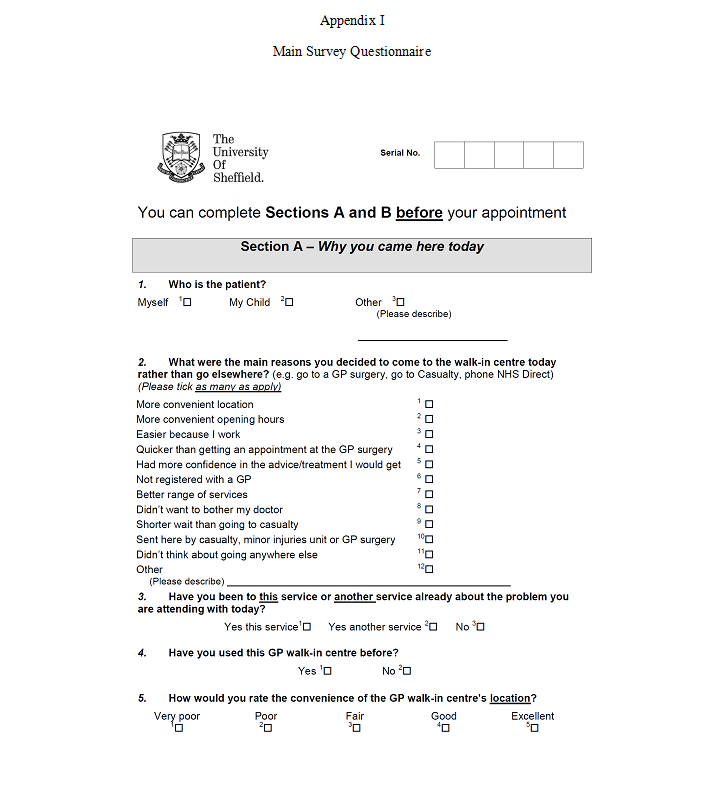

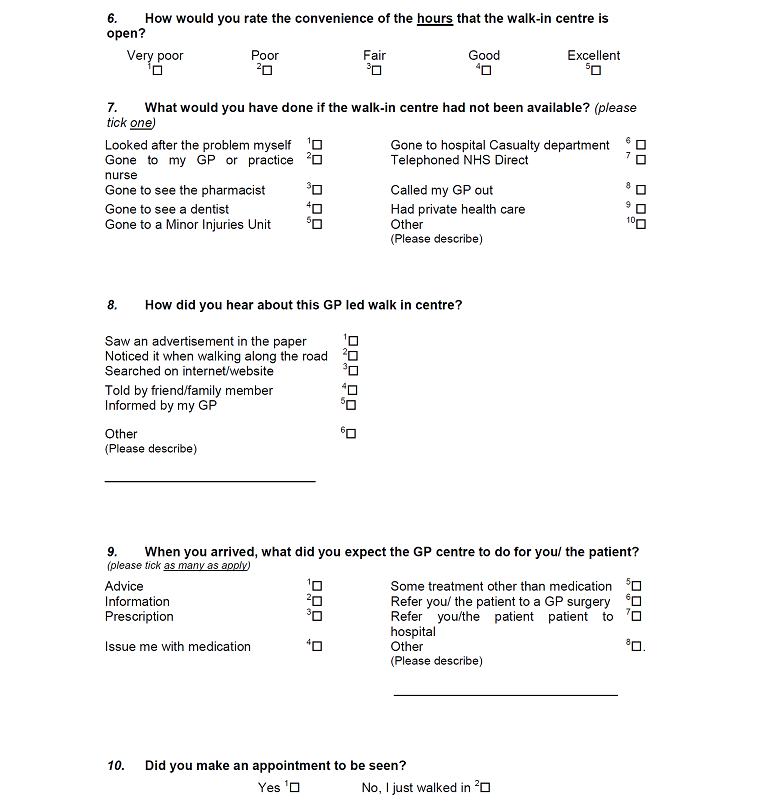

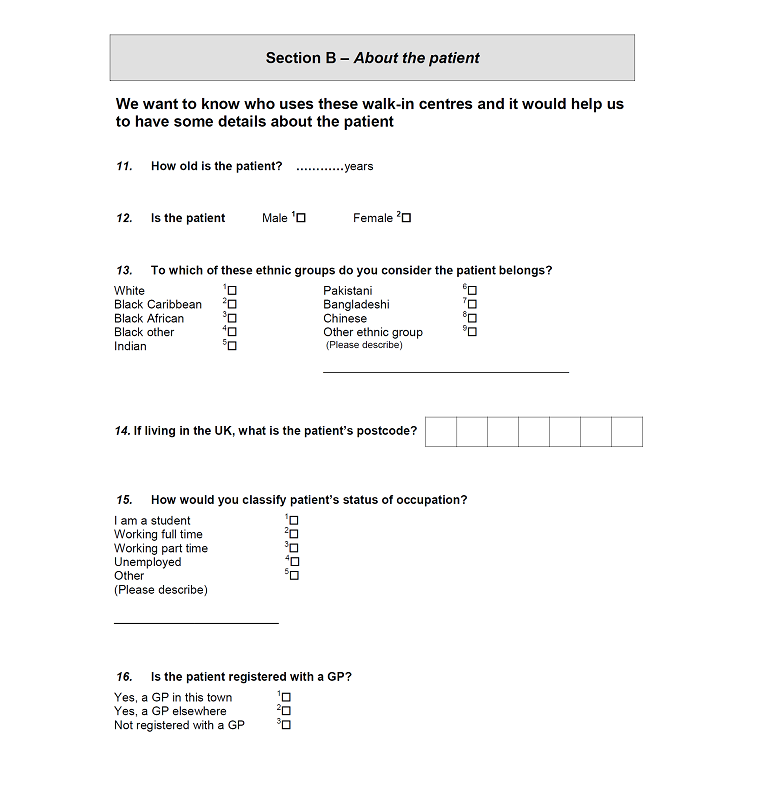

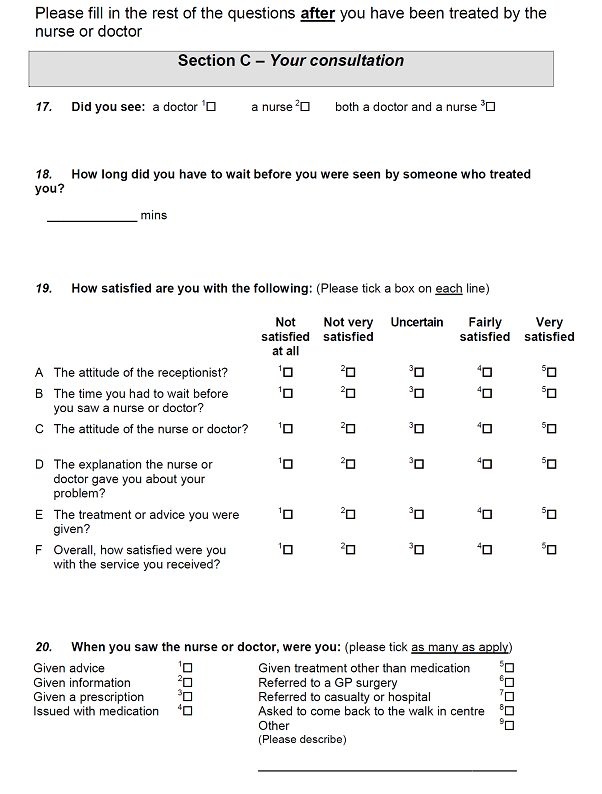

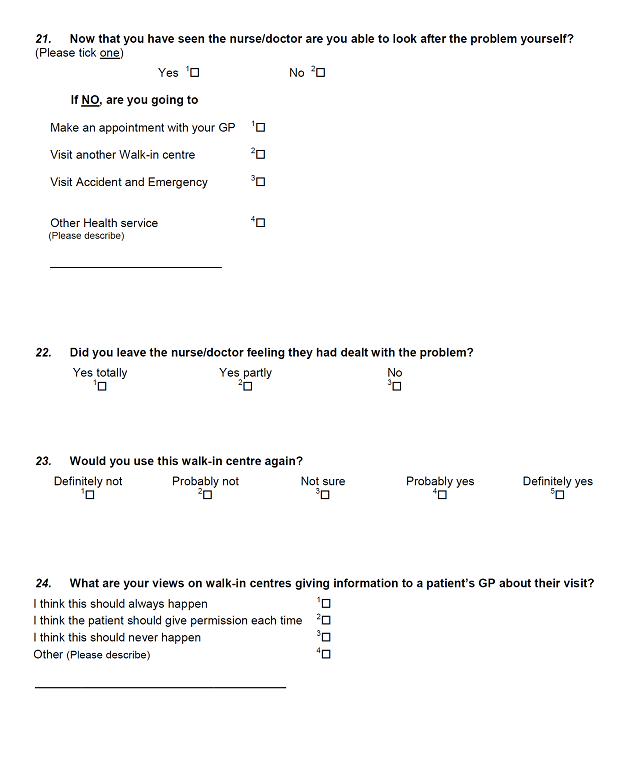

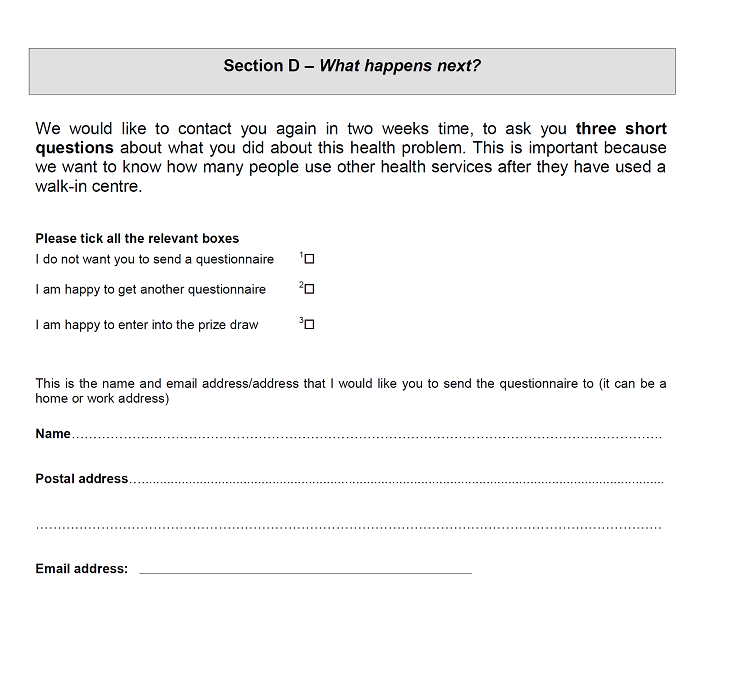

Supplement: Additional file 1 — Appendix I. Main Survey Questionnaire. [file 1472-6963-13-142-S1.doc]

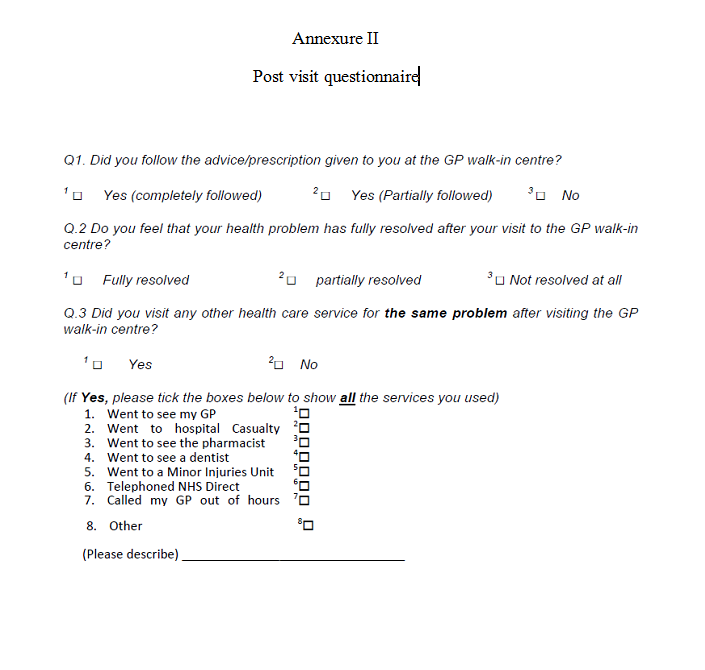

Supplement: Additional file 2 — Appendix II. Post visit questionnaire. [file 1472-6963-13-142-S2.doc]
